# Effect of Music Based Therapy Rhythmic Auditory Stimulation (RAS) Using Wearable Device in Rehabilitation of Neurological Patients: A Systematic Review

**DOI:** 10.3390/s23135933

**Published:** 2023-06-26

**Authors:** Sofia Scataglini, Zala Van Dyck, Véronique Declercq, Gitte Van Cleemput, Nele Struyf, Steven Truijen

**Affiliations:** 4D4ALL Lab, Department of Rehabilitation Sciences and Physiotherapy, Center for Health and Technology (CHaT), Faculty of Medicine and Health Sciences, University of Antwerp, 2000 Antwerpen, Belgium; zala.vandyck@student.uantwerpen.be (Z.V.D.); veronique.declercq@student.uantwerpen.be (V.D.); gitte.vancleemput@student.uantwerpen.be (G.V.C.); nele.struyf@uantwerpen.be (N.S.); steven.truijen@uantwerpen.be (S.T.)

**Keywords:** Parkinson’s disease, multiple sclerosis, stroke, spinal cord injuries, music-based therapy, rhythmic auditory stimulation (RAS), wearables

## Abstract

(1) Background: Even though music therapy is acknowledged to have positive benefits in neurology, there is still a lack of knowledge in the literature about the applicability of music treatments in clinical practice with a neurological population using wearable devices. (2) Methods: a systematic review was conducted following PRISMA 2020 guidelines on the 29 October 2022, searching in five databases: PubMed, PEDro, Medline, Web of Science, and Science Direct. (3) Results: A total of 2964 articles were found, including 413 from PubMed, 248 from Web of Science, 2110 from Science Direct, 163 from Medline, and none from PEDro. Duplicate entries, of which there were 1262, were eliminated. In the first screening phase, 1702 papers were screened for title and abstract. Subsequently, 1667 papers were removed, based on population, duplicate, outcome, and poor study design. Only 15 studies were considered after 35 papers had their full texts verified. Results showed significant values of spatiotemporal gait parameters in music-based therapy rhythmic auditory stimulation (RAS), including speed, stride length, cadence, and ROM. (4) Conclusions: The current findings confirm the value of music-based therapy RAS as a favorable and effective tool to implement in the health care system for the rehabilitation of patients with movement disorders.

## 1. Introduction

Due to an increase in neurological disorders in aging people, the economic burden on society is rising. Recent numbers mention an aggregated cost of 798 billion euros across Europe [1]. Brain injury, with an estimation of 60%, is the main cause of these numbers. However, other brain disorders, ranging from headaches to neuromuscular diseases and brain damage, also impose an economic burden [1]. To lower these expenses, we need to look for a suitable therapy for this expanding part of the population.

According to the systematic review of Sihvonen et al. [2], the need for a new cost-effective and easily applicable rehabilitation process is necessary. A possible solution is music therapy. Music is a multidisciplinary type of art and communication that forms a fundamental part of human society. Music is clinically used as an element of treatment in many disorders that involve affective, attention, memory, communication, or motor deficits [3]. The therapy, which involves listening to music or playing an instrument, improves neural connectivity between different brain areas [4]. This is because brain activity is increased due to sensory and cognitive stimulation. Hutchinson et al. [5] state that the motor and auditory brain are linked in neural networks that include both cortical and subcortical structures. This suggests that it may be possible to improve the motor deficits of neurological disorders by utilizing auditory stimulation. Music-based therapy is an example of auditory stimulation, as well as Rhythmic Auditory Stimulation (RAS) [6].

RAS is defined as a therapeutic application of pulsed rhythmic or musical stimulation to improve gait or gait-related aspects of movement [6]. It is a mode of facilitation that is often used in a neurological population, more specifically with patients suffering from Parkinson’s disease (PD). By utilizing RAS, a patient is more attentive while participating in an exercise augmenting her/his performances [7]. 

PD is a complex, age-related, neurodegenerative disease associated with dopamine deficiency and both motor and non-motor deficits, characterized by tremor, rigidity, and bradykinesia [8]. Persons with PD often experience freezing of gait, an abnormal gait pattern in which there are sudden, short, and temporary episodes of an inability to move the feet forward, despite the intention to walk [9]. Multiple sclerosis (MS) is a chronic inflammatory disease of the central nervous system which causes neurologic dysfunction, muscle weakness, unbalance and other conditions, such as fatigue, pain, depression, anxiety [10,11]. Spinal cord injury (SCI) is a term that refers to damage to the spinal cord as a result of trauma or from disease or degeneration. The severity of injury and its location on the spinal cord defines the symptoms. These may include partial or complete loss of sensory function or motor control of arms, legs, and/or body. The most severe SCI affects the systems that regulate breathing, bowel or bladder control, blood pressure, and heart rate. Chronic pain is experienced by most people with SCI [12]. Stroke is a disease which affects the blockage of blood flow to the brain, and is divided into two types [13]: (1) ischemic stroke: when a vessel supplying blood to the brain is obstructed by a blood clot; and (2) hemorrhagic strokes: caused by a weakened vessel that ruptures and bleeds into the surrounding brain. In this case, the blood accumulates and compresses the surrounding brain tissue [13]. Depending on how long the brain lacks blood flow, and which part is affected, a stroke can cause temporary or permanent disabilities [13]. Possible complications are: the difficulty talking or swallowing, memory loss or thinking difficulties, emotional problems, pain, changes in behavior and self-care ability, and paralysis or loss of muscle movement [14]. Systematic reviews have already been written on the effect of music therapy, or RAS, on neurological disorders, especially PD and stroke [15,16]. The systematic review about the potential benefits of music-playing in stroke upper limb motor rehabilitation, written by Grau-Sánchez et al. [15], examines the effects of actively self-playing an instrument to treat upper limb paresis to improve motor rehabilitation after stroke, improve cognition, engage patients, and increase motivation and well-being. Ashoori et al. [16], in their research, show that music not only helps in recovery after stroke, but can also improve walking in individuals with PD. They described the effects of rhythmic music stimulation and sound and how they may contribute to the improvement of motor disorders in various movement disorders [16]. The relationship between rhythm and locomotion allows RAS to be applied clinically for gait rehabilitation in patients with neurological disorders, particularly for the freezing of gait in patients with PD.

For music interventions and/or RAS to be integrated into clinical practice, the number of published studies and their level of evidence must be increased. The other crucial aspect is the magnitude of the treatment effect of music therapy and/or RAS. However, the usefulness and effectiveness of these therapies have already been proven, but there is conflicting evidence about their added value in rehabilitation therapy using wearable devices. Implementation is a major challenge, as aspects such as practicality, adaptation, and integration of these therapies into rehabilitation programs and centers must be considered. Implementing music-based interventions requires an analysis of the resources needed, including space, materials, and therapeutic requirements.

The information that is returned from the wearable can be used in the analysis of the patient’s gait pattern. This analysis consists of spatiotemporal parameters, such as walking speed, stride time, stride length and cadence, and their deviations [7]. Based on this preliminary evaluation, the intention of the systematic review is to understand the potential that music-based therapy has for neurological patients, hopefully sourcing the cost-effective and easily applicable rehabilitation process that is desired. For this reason, the aim of this systematic review is to investigate the effectiveness of music-based therapy RAS using wearable devices on a neurological population such as those with PD, stroke, MS, and SCI, focusing on balance and gait.

## 2. Materials and Methods

This systematic review is written according to the preferred reporting items for Systematic reviews and Meta-Analysis (PRISMA 2020) guidelines [17].

### 2.1. Eligibility Criteria

Studies where music therapy or RAS was performed on a neurological population were included. This neurological population included people with PD, stroke, MS, or SCI. Studies based on rehabilitation were included. Systematic reviews were excluded. There were no other restrictions imposed on study design. There were no restrictions imposed on the publication date of the studies. Studies had to be written in English or Dutch. It is important to note that there was no comparison with another intervention or population included. However, if a control group was present, we always included the article. We included not only RCTs, but also feasibility studies, to be able to include a larger selection of studies. A summary of the eligibility criteria following the PICOS method can be found in Table 1.

### 2.2. Information Sources

Studies were identified by searching four electronic databases. These were PubMed (https://pubmed.ncbi.nlm.nih.gov/ (accessed on 29 October 2022)), Web of Science (https://www.webofknowledge.com (accessed on 29 October 2022)), Medline (https://www.nlm.nih.gov/medline/medline_overview.html (accessed on 29 October 2022)), PEDro (https://pedro.org.au (accessed on 29 October 2022)), and Science Direct (https://www.sciencedirect.com (accessed on 29 October 2022)). The last search run was performed on 29 October 2022. Studies were downloaded into Endnote X9 (https://endnote.com/, accessed on 29 October 2022).

### 2.3. Search Strategies

Each database was searched using key word combinations. Search terms related to a neurological population, music therapy, RAS, and rehabilitation were included for identification of relevant articles. The full search strategy was developed in PubMed (Table 2) by using the Boolean operators ‘AND’ and ‘OR’, with the free keywords and MeSH terms from the PICOS method.

### 2.4. Study Selection

Titles and abstracts of potentially relevant articles were reviewed independently by three examiners (VD, GV, and ZV). Eligibility assessment was performed in a double-blind, standardized manner. The screening process consisted of two phases (phase 1: title and abstract; phase 2: full text), and each reviewer screened every item. Disagreements between reviewers were resolved by consensus. Full text articles of the eligibility studies were retrieved for further research. After the full text screening, the reviewers met to discuss which articles were suitable for inclusion and exclusion. Fifteen studies were included in the final review. The applied order of exclusion in chronological order was study participants with no neurological disorder, no music therapy, no RAS, no rehabilitation was reported, the study was a systematic review, and the study was not written in English or Dutch.

### 2.5. Data Collection Process

**Figure 1:**
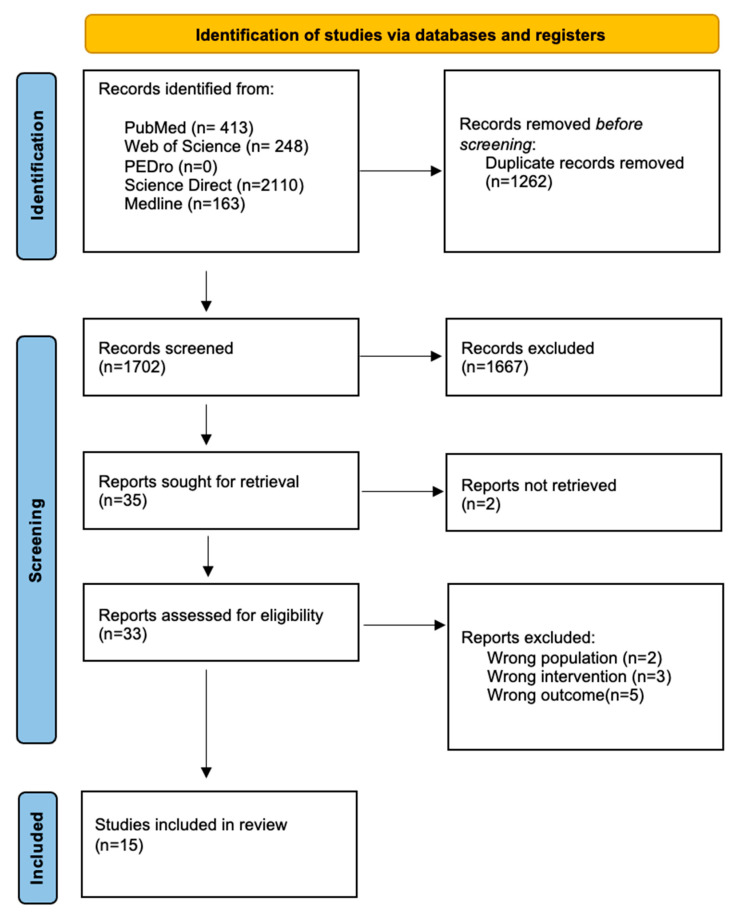
PRISMA 2020 flow diagram.

The collection of data was performed independently by the three reviewers. Fifteen studies (Figure 1) were distributed among the three researchers. Risk of bias of individual studies were made (Table 3 and Table 4). A detail description of these studies were collected in an evidence table (Table 5).

### 2.6. Data Items

Information was extracted from each included study on: (1) characteristics of trial participants (total number, distribution of men and women, and age); (2) type of intervention; (3) examination protocol; (4) outcome measures; (5) mean results.

### 2.7. Methodological Quality in Individual Studies 

Quality assessment was performed in relevant articles and was reviewed independently by all reviewers (sensors). Eligibility assessment was performed in a blinded, standardized manner. Any disagreements were resolved by further review of studies until consensus was reached. The ROB 2 tool is a revised Cochrane risk-of-bias tool for randomized trials, used to determine risk of bias of each article representing the methodological quality of the individual studies. The ROBINS-I tool was used to determine the risk of bias of non-randomized studies of interventions. The scale consists of a fixed set of domains of bias, focusing on different aspects of trial design, conduct, and reporting. Within each domain, a series of questions aim to elicit information about features of the trail that are relevant to risk of bias. The different domains are bias due to the randomization process, bias due to deviations from the intended interventions, bias due to missing outcome data, bias in the measurement of the outcome, and bias in the selection of reported outcomes. An algorithm generates a proposed judgment of bias risk from each domain based on the responses to the signaling questions. The judgment may be “Low”, “Moderate”, or “High” risk of bias, or may indicate “Some” concern. Studies were given a 🟩 on the specific domain of the bias risk assessment, indicating a good quality result. A 🟧 was assigned when the quality was low, and 🟥 if the quality was moderate. To determine the risk of bias of the case study, the Newcastle-Ottawa Scale (NOS) was used. This assessment scale consists of three domains: selection, comparability, and outcome. A maximum of one star for each numbered item within the selection and outcome domain can be given to a study. For comparability, a maximum of two stars can be given.

## 3. Results

We present the results from studies used for our systematic review in different sections, to create a clear overview. The order is presented as such: selection of studies, risk of bias, characteristics of individual studies, and results of individual studies. We used five databases: PubMed, Web of Science, Science Direct, Medline, and PEDro. The search query resulted in 2964 hits, including 413 articles from PubMed, 248 from Web of Science, 2110 from Science Direct, 163 from Medline, and none from PEDro. By using Endnote X9, 1262 duplicates were removed. In the first screening phase, 1702 articles were screened for title and abstract. As a result, 1667 articles were excluded, based on population, duplicate, outcome, and incorrect study design. Finally, 35 articles were screened for full text in the second phase. After the second screening, two articles were excluded based on wrong population, four based on wrong intervention, five based on wrong outcome, and seven based on wrong study design. Figure 1 shows the PRISMA 2020 [17] flow diagram summarizing the selection process and reasons for exclusion of the studies. A total of fifteen articles were considered eligible for this systematic review, nine non-randomized clinical trial studies and five randomized controlled trial studies.

### 3.1. Risk of Bias of Individual Studies

Table 3 provides an overview of the risk of bias assessment. The risk of bias was performed triple-blind between the investigators GV, VD, ZV. There was a small disagreement in the article [18] about the bias due to selection of reported results (91.67% agreement). We reviewed the article of disagreement together during an online meeting, discussed our differences, and explained to each other what our views were, which gave a different perspective on the issue of disagreement, and we came to a consensus. Five randomized controlled trials [19,20,21,22] were included in the review. These five articles were evaluated using the ROB 2 tools, a Cochrane risk-of-bias tool. Based on this tool, Refs. [19,20,21] received a low risk of bias (green square) in the five different domains, such as: bias due to randomization, bias due to deviations from intended intervention, bias due to missing data, bias in outcome measurement, and bias in selection of the reported result. Because the five domains had a low risk, we can conclude that the articles have a good quality result. Zhao et al. [22] scored a low risk of bias (green square) in all domains, except for the domain bias due to randomization, where it received a high risk of bias (red square). The study of Hankinson et al. [23] scored a high risk of bias due to deviations from the intended intervention. On all the other domains, the risk of bias score was low. Therefore, a low quality was assigned to these studies. The remaining nine articles [18,24,25,26,27,28,29,30,31] are non-randomized controlled trials. To assess the risk of bias in the intervention, they were assessed using the ROBINS tool (RoB2). Seven studies [19,20,21,24,28,29,30] have achieved the best possible score in the five different domains, which is a low risk of bias (green square). This indicates that these four studies are of good quality. De Bartolo et al. [25] acquired a moderate score on bias due to randomization and bias due to missing data, resulting in a moderate overall score. Guimaraes et al. [18] rated a low risk of bias (green) on all three domains and scored moderate on the domains of bias arising from the randomization process and bias in selection of the reported results. This made the overall quality of the article moderate. Lopez et al. [27] obtained the worst score of all the articles, scoring only low on bias in the outcome measurement and bias in selection of the reported results (green), poor on bias due to randomization process (red), and moderate on bias due to deviations from the intended intervention and bias due to missing data (orange). This made the overall quality of the article low. Kang et al. [26] and Yi Cai et al. [31] obtained moderate quality for their articles because of a moderate score on bias due to randomization. On all four other domains, these articles gained low risk.

**Table 3:** Risk of bias assessment–RoB2 tool.

Study	D1	D2	D3	D4	D5	Overall
De Bartolo et al. [25]	🟧	🟩	🟧	🟩	🟩	🟧
Cochen De Cock et al. [24]	🟩	🟩	🟩	🟩	🟩	🟩
Guimaraes et al. [18]	🟧	🟩	🟩	🟩	🟧	🟧
Lopez et al. [27]	🟥	🟧	🟧	🟩	🟩	🟥
Moumdjian et al. [28]	🟩	🟩	🟩	🟩	🟩	🟩
Uchitomi et al., 2013 [21]	🟩	🟩	🟩	🟩	🟩	🟩
Zhao et al. [22]	🟥	🟩	🟩	🟩	🟩	🟥/🟧
Uchitomi et al., 2016 [30]	🟩	🟩	🟩	🟩	🟩	🟩
Ginis et al. [20]	🟩	🟩	🟩	🟩	🟩	🟩
Erra et al. [19]	🟩	🟩	🟩	🟩	🟩	🟩
Park et al. [29]	🟩	🟩	🟩	🟩	🟩	🟩
Kang et al. [26]	🟧	🟩	🟩	🟩	🟩	🟧
Hankinson et al. [23]	🟩	🟥	🟩	🟩	🟩	🟥
Hi Cai et al. [31]	🟧	🟩	🟩	🟩	🟩	🟧

Legend risk of bias: D1 = Bias due to randomization; D2 = Bias due to deviations from intended intervention; D3 = Bias due to missing data; D4 = Bias in the outcome measurement; D5 = Bias in selection of the reported results; 🟩 = Low risk; 🟧 = Moderate risk; 🟥 = High risk.

The risk of bias of Kantan et al. [32], was determined using the Newcastle-Ottawa scale. The quality of each domain was scored, with a good quality for the selection domain and a poor quality for the comparability and outcome domain. This results in a high risk of bias. 

**Table 4:** Risk of bias assessment–NOS, each asterisk (*) represents the number of fulfilled criteria within the domain.

Newcastle-Ottawa Scale (NOS)					
	Domain			Risk	
Study	Selection	Comparability	Outcome	Score	Risk
Kantan et al. [32]	***		*	****	High

### 3.2. Study Characteristics

For each study, the characteristics for which data were retrieved are presented in Table 5.

#### 3.2.1. Population Characteristics 

Fifteen articles were included in this study. Of the fifteen included studies, six included both healthy controls and neurological diseases, such as PD [19,25,30,31], patients with stroke, and patients with MS [28] (N = 287, age range 27–82; N = 22, age range >18; N = 61, age range 40–64, respectively). Seven [18,20,21,22,24,27,29] of the fifteen included studies had patients with PD (N = 159, age range 45–78); two [26,27,28,29,30,31,32] of the fifteen included studies had patients with a hemiparetic stroke (N = 24, age range 34–66). See Table 5 for a detailed overview of the descriptive characteristics of the participants across studies.

**Table 5:** Evidence table (6MWT: Six-Minute Walk Test, AI: Asymmetry Index, APDM system: Ambulatory Parkinson’s Disease Monitoring system, ASV: Arm Swing Velocity, CG: Control Group, ConCue: Continuous Cueing, CoVSwing: The coefficient of variation of the swing, CVA: Cerebrovascular Accident, DS: Double Support, FMA: Fugl-Meyer Assessment, FOG: Freezing of Gait, GPQI: Kinematic Gait Phase Quality Index, H&Y: Hoehn and Yahr stage, HC: Healthy Control, ICG: Interaction Condition Group, IMU: Inertial Measurement Units, IntCue: Intelligent Cueing, IntFB: Intelligent Feedback, LL: Lower Limb, LR: Loading Response, MAC: Melodic Auditory Cueing, MS: Multiple Sclerosis, MT: Movement Time, MV: Movement Variability, NAC: No Auditory Cueing, NoInfo: No Information, O: Obliquit, PD: Parkinson’s Disease, POSTCG: Post-interaction Condition Group, PRECG: Pre-interaction Condition Group, PW: Preferred Walking, PwMS: Person with Multiple Sclerosis, R: Rotation, RAS: Rhythmic Auditory Stimulation, RAS90: RAS at 90% of PW, RAS100: RAS at 100% of PW, RAS110: RAS at 110% of PW, RCT: Randomized Controlled Trial, ROM: Range of Motion, rPA: Average relative Phase Angle, RVL: Resultant Vector Length, SCI: Spinal Cord Injuries, SD: Stride Duration, SI: Symmetry Index, SL: Stride Length, St: Stance Phase, Sw: Swing Phase, T: Tilt, UL: Upper Limb, ↑ WS: walking speeds in m/s; ↓ Decrease).

Reference	Participants Characteristics	Intervention Details	Wearable Device	Procedure of Examination Protocol	Outcome Measures	Main Results
Study Design	N = Population g = (m/f)					
	Age (Years)					
Music-Based Therapy						
De Bartolo et al., 2019 [25]	PD	-Classic music (andante and allegro)	YES	14 trials for each subject	Spatiotemporal gait parameters (WS, SL, SD, St, Sw, DS)	Difference in spatiotemporal parameters among different musical conditions
	N = 20 (14/7)	-Pop music				
Non-randomized trial	72.5 ± 9.2 years	-Rock music (motivational hard rock and arena rock)	Wireless headphone	1 trial; no music (NM)	Pelvis kinematics	WS ↑: classical to heavy metal music
		-Heavy metal music		6 trials; random music tracks	(T-ROM, T-SI, R-ROM, R-SI, O-ROM, O-SI)	
	Elderly adults		Wearable IMU	7 trials in reverse order finishing with NM trial		The main effect of the group of subjects was found significantly for speed (*p* < 0.001), stride length (*p* < 0.001), the three ranges of motion (*p* = 0.002), rotation (*p* = 0.014), and obliquity symmetry indices
	N = 20 (8/12)	ON-medication	(Ergonomic waist belt at the level of the sacral vertebrae S1, S2)			
	72.1 ± 5.6 years					
	Young adults					
	N = 20 (8/12)					
	32.3 ± 5.93 years					
Cochen De Cock et al., 2021 [24]	PD (H&Y 2.4 ± 0.5)	Patient chooses 2 genres/session	YES	30 min walking listening to music (4w; 5x/w)	Gait measurements (distance, cadence, velocity, SL and AI) before and after 4-week rehabilitation program	↑ distance (*p* = 0.01), ↑ cadence (*p* = 0.01), ↑ speed (*p* < 0.01), and ↑ stride length (*p* = 0.04)
Non-randomized clinical trial	N = 45 (25/20)					
	65 ± 9 years	Disco	BeatWalk (smartphone application)	Gait parameters: 6MWT via sensors (IMU)	Safety and tolerance	Falls: during rehab < before (*p* = 0.07)
		Soft pop				Pain: during BeatWalk < baseline (*p* = 0.02)
		Pop rock	Ankle-worn sensors, and sternum sensor		Training program observance, usability, and enjoyment	Fatigue: during BeatWalk = baseline, on visual sliding scale (*p* = 0.3), and on fatigue severity scale (*p* = 0.5)
		Instrumental				
		Variety			Physical activity evaluation	# Responses: positive > negative (*p* < 0.001)
		The program applied online stimulus adaptation (time stretching algorithm)				
		ON-medication				
Park et al., 2021 [29]	PD (H&Y 1–3)	Familiar and unfamiliar music cues (90–120 bpm)	YES	2 min walking w/pivoting	Spatiotemporal gait parameters (cadence, SD, gait velocity, SL, ASV, and duration and the coefficient of variation)	Entrained walking with either familiar or unfamiliar music cues compared with baseline
	N = 20 (13/7)					↑: gait velocity (*p* < 0.01), SD, and/or cadence (*p* < 0.05), SL (*p* > 0.10), ASV (*p* < 0.001), and arm swing ROM (*p* < 0.001)
Non-randomized clinical trial	68.85 ± 5.39 years	ON-medication	APDM system	Baseline (no cue)	20-point VAS (perceived familiarity, cognitive demand, physical demand, beat salience, and enjoyment)	↑ variability of stride-to-stride time (*p* < 0.05) and length
			6 wearable sensors/IMU (feet, wrist, waist, sternum)	2 sessions (familiar and unfamiliar in random order), with 5 min break between sessions		
			Headset			Unfamiliar < familiar music: SL (*p* > 0.10), enjoyment (*p* < 0.001), and beat salience (*p* < 0.01)
						Walking with unfamiliar and familiar music cues: ↑ stride amplitude and ↑ arm swing amplitude (*p*’s < 0.01) of PD
Hankinson et al., 2022 [23]	Sub-acute stroke	Individualized music-motor therapy (BPM)	YES	Baseline	FMA UL & LL	UL scores improved in both groups at 3-week follow-up
	N = 22 (13/9)			3 weeks		
Single blind RCT	>18 years	20 min 3x/week	GotRhythm	6 weeks		
		GotRhythm Intervention: warm-up, preparatory activities (BPM/movement selection), main activities and cool down	IMU (on relevant body part)			
		Control: usual care, no additional music-motor therapy				
Kantan et al., 2022 [32]	Subacute stroke patients, with predominantly one-sided weakness	Music sonification strategies	YES	Formative and summative evaluations (following the second development cycle)	Balance (spatial)	The interactions of the studies were deemed to be meaningful and relevant to clinical protocols, with comprehensible feedback for several patient types
	N = 6 (4/2)	Continuous or discrete MBF strategy			Sit-to-stand	
Case study			Single IMU sensor: lower back		Gait training	System benchmarking:
			Pair of IMU sensors: ankles		Trunk accelerations/sway velocities	-Sufficiently short loop delays: ca. 90 ms
						-Healthy sensing range: >9 m
			Monophonic loudspeaker placed in the room			-Computationally efficient: 11.1% peak CPU usage on a quad score processor
Rhythmic Auditory Stimulation (RAS)						
Guimaraes et al., 2015 [18]	PD (H&Y 2.4 ± 0.3)	Metronome sounds	NO	20-m walking test	Activations and deactivations of cueing	Between non-cued and cued walking tests, there were no significant differences in walking speed (*p* = 0.49), step length (*p* = 0.75), and cadence (*p* = 0.29)
	N = 12 (7/5)	Musical beats			Applied cueing rhythm	There was a significant difference between applied and measured rhythms (*p* < 0.01)
Non-randomized trial	71.2 ± 1.4 years	Clapping	Headset connected to 2 smartphones (walking rhythm detector & gait problems detector)	2x non-cued	Walking rhythm	
		Verbal cueing		2x cued	Type of impairment detected	
					The adjustments of rhythm performed by the therapist in real time	
Lopez et al., 2014 [27]	PD (H&Y 2.5–3)	Metronome	YES	Gait Analysis Laboratory with reflective markers for 3D video data	Time to complete walkway (s)	Walking speed, cadence, and stride length: ON > OFF (*p* = 0.0117)
	N= 10 (7/3)					
Non-randomized trial	45–65 years	OFF-dopamine	Listenmee^®^, a portable device	7.62-m walkway	Average walking speed was calculated (m/s)	All patients improved in the motion analysis
				2x baseline without Listenmee^®^	Average cadence (steps/min)	
				1x with Listenmee^®^ without RAS (OFF)		
				1x with Listenmee^®^ with RAS (ON)	Average stride length in meters (distance/number of steps)	
					Mean of 2 trials (device on/off), this was used for the analysis	
Uchitomi et al., 2013 [21]	PD (H&Y 2.44 ± 0.520)	Interactive Walk-Mate	YES	1 trial = 200 m	Baseline gait performance	Baseline walking trial fractal scaling exponent, mean and coefficient of variation of stride intervals: =for all groups (*p* > 0.1)
	N = 32 (18/14)	Fixed tempo				
RCT	70.4 ± 8.24 y	1/f fluctuation tempo	Walk-Mate system	3 trials/day (4days)	Gait-relearning effect (fractal scaling of stride intervals, mean and coefficient of variation for stride intervals)	The gait-relearning effect γ_f_ of the fractal scaling exponent of the stride interval:
		Silent control	Headphones	1x baseline		Walk-Mate > fixed tempo, 1/f fluctuation tempo and silent control (*p* < 0.05). No differences between the silent control group, the fixed tempo group, and the 1/f fluctuating tempo group (*p* > 0.05)
			Pressure sensors (shoes)	2x rhythmic cue	Interpersonal synchronization between steps and cues	
		ON-medication				The mean γ_m_ and coefficient of variation γ_c_ of stride intervals did not differ significantly between all the four conditions (resp. *p* = 0.3 and *p* = 0.2)
						The stride intervals of the PD subjects in the fixed tempo group and the 1/f fluctuating tempo group did not synchronize (*p* > 0.1), and, in the interactive Walk-Mate group, they were synchronized (*p* < 0.01)
						The mean of the circular variance:
						Walk-Mate < fixed tempo and 1/f fluctuating tempo (*p* < 0.01)
Zhao et al., 2016 [22]	PD (H&Y 2–3)	Metronome	YES	4 walking courses	FOG number and duration	FOG number and duration
RCT	N = 12 (9/3)	LED			Stride length	FOG%: full turn > narrow turn (*p* < 0.05)
	66.8 ± 6.8 years	Optic flow	Google Glass	1. wide turn (180°)	Walking speed	FOG duration: no sign difference (*p* > 0.05)
				2. narrow turn (180°)	Cadence	Full: #FOG: metro < no cue (*p* < 0.05)
		End of medication dose	MVN motion capture suit with 7IMU (pelvis, upper legs, lower legs, and both feet)	3. full turn (180° + 360°)		
				4. doorway (2 m + 90° + doorway + 180°)		Stride length:
						Cues > no cues (*p* < 0.001)
			Video recordings anterior-posterior axis at start and midpoint			Metronome ↑ (*p* < 0.05)
						Optic flow ↓ (*p* < 0.01)
						LED ↓ (*p* < 0.005)
						Walking speed:
						Doorway: metro > no cue (*p* < 0.05)
						Wide
						ptic ↓ (*p* < 0.01)
						ED ↓ (*p* < 0.001)
						Narrow
						ptic ↓ (*p* < 0.001)
						ED ↓ (*p* < 0.001)
						Cadence:
						Narrow: Cues ↓ vs. no cues
						-etro (*p* < 0.01)
						-ED (*p* < 0.001)
						-ptic flow (*p* < 0.001)
						Full: cues ↓ vs. no cues
Uchitomi et al., 2016 [30]	PD (H&Y 2.77 ± 0.45)	Interactive rhythmic cues	YES	80 m corridor	Rate of change of stride interval (β)	The experimental conditions affected rate β (*p* < 0.001)
	N = 30 (16/14)					PRECG < ICG, POSTCG, CG (*p* < 0.001)
Non-randomized controlled trial	74.9 ± 7.10 years		Walk-Mate system:	1. Preliminary task	Mean and coefficient of variance in stride interval	ICG < POSTCG (*p* = 0.3), CG (*p* = 0.06)
			Headphones, pressure sensors (bottom shoe), and PC	2. Experimental task with 3 conditions (pre-interaction, interaction, and post-interaction)		POSTCG < CG (*p* = 0.9)
	CG					
	N = 18 (12/6)			Cues were provided during the middle third of the walk and stopped for the last third		The experimental conditions did not affect the mean stride interval (*p* = 0.8)
	70.6 ± 3.07 years					
						The experimental conditions affected the coefficient of variation (*p* < 0.001)
						CG < PRECG, ICG, POSTCG (*p* < 0.01)
						Nonsignificant differences between PRECG, ICG and POSTCG within PD subjects (*p* > 0.1)
Ginis et al., 2017 [20]	PD (H&Y 1–3)	ConCue	YES	6 w, 4 × 30 min walk with 1 w interval	Gait deviations and	Deviations:
	N = 28	IntCue			Subjective preferences	FOG+ > FOG- during IntCue (*p* = 0.04) and IntFB (*p* = 0.03)
RCT		IntFB	Headphones	Elliptical trajectory: 24 × 9 m		
	FOG +	NoInfo	IMU (2xfoot)			FOG+: ConCue < NoInfo (*p* = 0.02) and IntFB (*p* = 0.01),
	N = 15 (14/1)					
	62.80 ± 6.91 years	ON-medication				IntCue and IntFB < NoInfo (*p* = 0.04 and *p* < 0.05, respectively) in FOG+
	FOG-					FOG−, no significant differences were found between the conditions.
	N = 13 (9/4)					
	61.15 ±7.08 years					Most participants preferred the IntCue and IntFB.
Erra et al., 2019 [19]	PD	RAS	YES	20 m pathway	Spatiotemporal parameters (step length, SL, cadence, WS, SD, ROM_Hip_, ROM_Knee_, ROM_Ankle_, LR, flat foot, Pre-Sw, and Sw)	G1: ON and OFF
	N = 30 (20/10)			Four conditions:		RAS90
RCT	72 ± 6 years	ON and OFF medication	4 force resistive sensors under each foot (heel, 1st MTP, 5th MTP, and toe)	-PW	GPQI	↓ cadence, WS, ST, LR (*p* < 0.01 in all cases) and flat foot (*p* = 0.02)
				0		↑ preSw (*p* = 0.01)
	HC		2 wireless modules (feet)	0	Post-hoc: 2 groups	
	N = 18			0	G1: entire cohort of patients with PD	RAS100
			7 IMU (feet, 2xlat on mid-shanks, 2xlat on mid-thighs and pelvis)		G2: patients presenting high level of gait impairment	↓ ST (*p* < 0.01)
						↑ preSw (*p* = 0.01)
						RAS110
						↑ preSw (*p* = 0.01) and GPQI (*p* = 0.04)
						G2: ON and OFF
						RAS90
						↑ step length (*p* < 0.01), SL (*p* < 0.01) and ST (*p* < 0.01)
						RAS100
						↑ step length (*p* < 0.01), SL (*p* < 0.01)
						↓ GPQI (*p* = 0.01)
						RAS110
						↓ ST (*p* < 0.01) and GPQI (*p* = 0.01)
						↑ step length (*p* < 0.01), SL (*p* < 0.01), cadence (*p* = 0.05) and WS (*p* = 0.02)
Kang et al., 2020 [26]	Hemiparetic stroke	NAC	YES	Abduction-holding-adduction trials	Spatiotemporal kinematic parameters (ROM, MV, MT and duration)	During holding phase, there existed a significant main effect of the cue on the ROM and MIN (*p* < 0.01)
	N = 18 (10/8)	RAS			Euler angle	ROM: NAC > MAC
RCT	2 were excluded	MAC	6 IMU (head, torso, arms, and forearms)	P1 = practice		MIN: MAC > NAC
	49.78 ± 15.55 years			P2 = experimental		
						Significant main effect of cue on the duration (*p* < 0.001)
						Holding duration: NAC > RAS > MAC
						RMSE: MAC < NAC, RAC
						Euler angle: RAC < MAC < NAC
Yi Cai et al., 2022 [31]	Idiopathic PD	-Unassisted silent control	YES	Safe walkway:	Gait parameters:	cooperative rhythmic stimulation:
Non-randomized controlled trial	N = 58 (36/22),	-Fixed tempo		-3 sets of climbing stairs. (Up and down)	-Gait cycle time	-stride time fractal scaling exponent ↑
	Mean 69.2 years	-Cooperative Rhythmic stimulation	Wrapped shoe based plantar pressure sensing device	-10 x Down and up -15 m ramp	-Swing time	-stride time fractal scaling exponent >fixed-tempo stimulation condition
		-Cooperative Rhythmic Stimulation with background musical track		-3 x walking 50 m	-Stance time	Stability in movement ↑with fixed tempo > the silent controls.
	Healthy control		Headphones		-Double support time	
	N = 73 (37/36)				-Stride length	
	Mean 67.4 years				-Step length	
					-Centre of pressure	
					-Hind pressure	
					-Mid pressure	
Music-based therapy and Rhythmic Auditory Stimulation (RAS)						
Moumdjian et al., 2019 [28]	PwMS	3 conditions	YES	12 min walk to 3 conditions and 15 min rest in-between	Primary outcome (rPA, RVL, perceived physical fatigue, perceived cognitive fatigue, and motivation)	All participants synchronized to both stimuli; however, PwMS synchronized better when walking to music, compared to metronomes (*p* = 0.0005)
Non-randomized controlled trial	N = 31 (8/23)	Music				
	53.45 ± 10.61 y	Metronomes	D-Jogger: adaptive music player		Secondary outcome (cadence, speed, and stride length)	Coupling ↓ in the last 3 min of walking to both stimuli (*p* = 0.0056)
		Silence				
	HC		Headphones			PwMS perceived cognitive fatigue
	N = 30 (8/22)					Music < metronomes and silence (*p* = 0.0002)
	51.77 ± 11.40 y		2 wireless IMU (ankles)			PwMS perceived physical fatigue
						= across the conditions (*p* = 0.0002)
			3 APDM (OPAL) wearable sensors (ankles and sternum)			
						Walking distance: GC > PwMS across all conditions
						HC cadence ↑ and got more into phase-locking: music and metronomes > silent PwMS slightly ↓ cadence

#### 3.2.2. Interventions

Regarding interventions, five studies evaluated the effects of music-based therapy [23,24,25,29,29,32], nine studied the effects of RAS [18,19,20,21,22,26,27,30,31], and, finally, the last article studied the effect of both interventions [28].

Six custom-made wearables were found in the fifteen included studies; these were the Beat-Walk [24], the Listenmee^®^ [27], the Walk-Mate [21], the Google Glass [22], the D-Jogger [28], and the GotRhythm [23]. A short description of these wearables can be found in Table 6.

#### 3.2.3. Motion Analysis Tools

The motion analysis tools that were used in the included articles were wearables and tools that includes also a combination of wearables devices and IoT devices. 

Inertial Measurement Units (IMU), a type of wearable device, were used in nine of the fifteen included articles [20,22,23,24,25,26,28,29,32]. They were used in the form of a waist-belt at the level of the sacral vertebrae S1, S2. IMUs were also applied around the pelvis area, head, torso, arms, forearms, upper legs, lower legs, ankles, and both feet. Another type of wearable device used were pressure sensors under the feet in two of the fifteen included articles [21,22,23,24,25,26,27,28,29,30]. They were used on the bottom of the shoes, as well as force resistive sensors that were placed underneath each foot (heel, 1st MTP, 5th MTP, and toes). Two of the fifteen included articles [19,20,21,22,23,24,25,26,27,28,29,30,31] used two types of wearable devices, both IMU and force-resistive sensors under each foot.

Two of the fifteen articles [18,19,20,21,22,23,24,25,26,27] did not use a wearable device. Guimaraes et al. [18] used two smartphones, one to detect the walking rhythm, and the other to detect gait problems (e.g., reduced speed, FOG, etc.). The detection was done by two therapists, one for each smartphone. The other study, Lopez et al. [27], used reflective markers and several cameras (video and infrared) to analyze the gait of the participants.

#### 3.2.4. Outcome Measures 

Most outcome measures concerned spatiotemporal gait parameters and gait kinematics (Table 7 and Table 8). The definition of each parameter and their units of measurement are mentioned in Table 7. While Table 8 summarizes the spatiotemporal and kinematic gait parameters measured across studies.

The gait-relearning effect was measured in two articles [21,22,23,24,25,26,27,28,29] of the fifteen included studies. They used the coefficient of variation; this was calculated for stride-to-stride variability and the fractal scaling of stride intervals. The interpersonal synchronization between steps and cues was evaluated to determine the capacity to synchronize cues in the study of Uchitomi et al., 2013 [21].

Park et al. [29] also evaluated the perceived familiarity, cognitive demand, physical demand, beat salience, and enjoyment using 20-point visual analogue scales. Perceived physical and cognitive fatigue and motivation was evaluated in the study of Moumdjian et al. [28], using a 10-point visual analogue scale and a Likert scale of 1–5, respectively.

The change in Fugl-Meyer assessment scores was measured in the article of Hankinson et al. [23]. This is a stroke-specific, performance-based impairment index that evaluates the motor function of both upper and lower limbs, as well as balance, sensation, and joint functioning. Items measuring movement, speed and coordination, and reflex actions of the shoulder, elbow, forearm, wrist, hand, hip, knee, and ankle are included in the motor domain. Items are scored on an ordinal scale of 0 (cannot perform, absent), 1 (partial impairment), and 2 (no impairment). There is a range from 0 (hemiplegia) to a maximum of 100 (normal motor performance) for the overall motor scores, with 66 points assigned for the upper extremity and 34 points for the lower extremity.

In the study of Kantan et al. [32], the system computed three different parameters; the trunk inclination angles, the mean-squared jerk, and the foot-strike detection. The first, trunk inclination angles, is calculated using accelerometer and gyroscope readings. Mean-squared jerk can capture instantaneous intermittencies with sufficient speed and sensitivity for concurrent feedback on smoothness. The last parameter, foot-strike detection, is measured using ankle sensors. 

### 3.3. Results of Individuals Studies

For this systematic review, we chose fifteen papers and grouped them into three main categories. The first group consists of five papers [23,24,25,29,32] that were categorized based on music-based outcomes, nine articles [18,19,20,21,22,26,27,30,31] that used RAS, and one article [28] which combined music-based therapy with RAS. A summary of each individual study is included in Table 5.

#### 3.3.1. Music Therapy-Based Results

The following five articles [23,24,25,29,32] investigated the effects of music therapy on movement outcomes. 

The study by De Bartolo et al. [25] aimed to analyze gait variability between different musical conditions in spatiotemporal parameters and trunk oscillations. In this study, 20 patients with PD (PP group: mean age 72.5 ± 9.2 years; 14 M, 6 F), 20 older adults (EA group: mean age 72.1 ± 5.6 years, 8 M, 12 F), and 20 young adults (YA group: mean age 32.3 ± 5.93 years; 8 M, 12 F) participated. The procedure consisted of measuring the different gait parameters while the patient listened to different types of music with portable headphones. The music genres ranged from classical, pop and rock to heavy metal music. Gait data was collected using a portable IMU, G-Walk, BTS, OR Padua, provided with a triaxial accelerometer, a triaxial gyroscope, and a triaxial magnetometer. The device was worn with a hip belt at the level of the sacral vertebrae S1–S2, connected to a portable computer via Bluetooth. 

The main effect of music tracks on the values of spatiotemporal gait parameters and ROM of the trunk were found to be statistically significant in all three groups: speed (*p* < 0.001), stride length (*p* < 0.001), and for the three ranges of motion (*p* = 0.002) and rotation (*p* = 0.014). However, the values of the symmetry indices were not statistically significant. Post hoc analyses were conducted to identify the main effects of music and to highlight the effects of different types of music. This showed a reduced speed (*p* < 0.001), rotation (*p* = 0.014), tilt ROM (*p* = 0.002), and increased stride time (*p* < 0.001) in the classical Andante (Chopin’s prelude). In Classical Allegro (Beethoven’s Ninth Symphony), a similar effect was noted with a significant reduction in velocity (*p* < 0.001) and tilt ROM (*p* = 0.001), and an increase in stride duration (*p* < 0.001). ROM also tended to result in a diminution by pop music (*p* = 0.005); conversely, it was increased by rock (*p* = 0.009), motivational (*p* < 0.001), and heavy metal (*p* = 0.014) songs.

Forty-five patients suffering from PD (age 65 ± 9; 25 men) participated in the study of Cochen De Cock et al. [24]. These patients exhibited gait disturbances but were able to walk independently. In this study [24], the aim was to evaluate the compliance, safety, tolerance, usability, and enjoyment of a new smartphone application. This application was attached to wearable sensors on the ankle and sternum (BeatWalk) and delivered individualized musical stimulation for automatic rehabilitation of gait at home. The music tempo was adjusted in real time to the gait frequency of patients, in a way that could promote an increase of up to +10% in their spontaneous cadence. Both neurological and neuropsychological assessments and gait measurements were performed before and after the 4-week rehabilitation program. Each patient was assessed at the same time of day, one hour after taking their medication. Spatiotemporal gait parameters were then recorded via sensors (inertial measurement units, including 3D accelerometers and gyroscopes, Mobility Lab, APDM Inc., Portland) which were strapped across the feet and the anterior side of the left and right tibia and sternum during the six-minute gait test. 

The percentage of patients who used the application for the prescribed duration was 78.8%. There was a significant improvement in distance (*p* = 0.01), cadence (*p* = 0.01), speed (*p* < 0.01), and stride length (*p* = 0.04) of the six-min walk test, taken pre-and post-intervention. 

Park et al. [29] investigated whether familiar and unfamiliar music cueing differentially influences stride and arm swing amplitude and stride-to-stride variability. They also investigated how stride and arm swing amplitude and stride-to-stride variability is altered by enhanced familiarity with music by repeated listening and walking during rhythmically cued walking in persons with PD.

Twenty individuals with idiopathic PD (mean age = 68.9 years, 7 females, H&Y stage 1–3) walked in time with familiar and unfamiliar music cues provided by a headset. They then repeatedly listened and walked to the same familiar and unfamiliar music cues. Spatiotemporal gait parameters in each 2-min trial were recorded with motion capture wearable sensors. The APDM system consists of 6 wearable sensors. These sensors were placed at the feet, wrist, waist, and sternum.

First results gave faster gait velocity (*p* < 0.01), stride time, and/or cadence (*p* < 0.05), greater stride length (*p* > 0.10), arm swing peak velocity (*p* < 0.001), and arm swing ROM (*p* < 0.001), as well as higher variability of stride-to-stride time (*p* < 0.05) and length compared with the baseline. Compared with familiar music, stride length (*p* > 0.10), enjoyment (*p* < 0.001), and beat salience (*p* < 0.01) were greater when walking with familiar music cues. Walking with music cues, even unfamiliar music, immediately enhanced stride amplitude and improved arm swing amplitude (*p* < 0.01) in PD.

Hankinson et al. [23] opted for a customized music-motor therapy and real-time biofeedback cell phone app used to promote rehabilitation after stroke. Twenty-two stroke survivors, who came from a sub-acute rehabilitation unit, participated in the study. All the participants received GotRhythm training, which was tailored to a participant’s specific injury. GotRhythm is a mobile phone-based app used to deliver individualized music-motor therapy. GotRhythm supports a wide range of sensors to sense a variety of physical activities, including movements of both upper and lower limbs and gross and fine motor skills. Based on the findings, we conclude that UL scores improved in both groups at a 3-week follow-up.

Kantan et al. [32] aimed to research musical biofeedback in poststroke movement rehabilitation. Six subacute stroke patients (four men and two women) volunteered to participate. They selected a distributed biofeedback structure with portable wireless inertial sensors and remote processing on a laptop. Depending on a patient’s case, there is either a single IMU sensor strapped to the patient’s lower back, or a pair of sensors strapped to the patient’s ankles. The music generation was accomplished by a system that generates an eight-track stereo instrumental ensemble that contains melodic and percussive elements in a 4/4-time signature. Findings established that the system has sufficiently short gait delays (90 ms) and a healthy detection range (>9 m). However, future studies will need to focus on using this framework with patients to both further develop its interactions and measure its effects on motor learning, performance retention, and psychological factors to gauge its true clinical potential.

#### 3.3.2. Rhythmic Auditory Stimulation (RAS)

The effects of RAS on the rehabilitation of neurological patients were investigated in the following nine articles [18,19,20,21,22,26,27,30,31].

Twelve people (7 men, 5 women, mean age 71.2 ± 1.4 years), diagnosed with PD and able to walk independently, participated in the study by Guimaraes et al. [18]. They researched the development of an auditory cueing system to assist gait in patients with PD. This work utilized a system that was developed and tested to provide real-time auditory stimuli through a headset connected to a smartphone. These stimuli were provided when certain episodes of change in speed and amplitude of movements were identified. Thus, they aim to investigate the feasibility of the system in stimulating walking using self-adaptive cueing rhythms to evaluate the benefits of its integration.

The results indicated there were no significant differences in walking speed (*p* = 0.49), step length (*p* = 0.75), and cadence (*p* = 0.29) between non-cued and cued walking tests. There was a significant difference between applied and measured rhythms (*p* < 0.01). Despite the results not being statistically significant, a trend could be noted to value cued walking higher than non-cued walking performance. As such, all participants would be willing to use the system during their daily lives because it helps them perform activities of daily living.

Lopez et al. [27] analyzed the effect on gait using Listenmee^®^. The present study included 10 patients with idiopathic PD who presented with gait disturbances, including freezing. Auditory rhythmic cues were delivered through Listenmee^®^, a portable auditory device with a 64 GB SD card that can produce 100 different sounds that the user can download on the device or app. The device also has statistical analysis software that can measure changes in distance and time. The data from the device can be accessed by physicians through the internet. Performance was analyzed in a motion and gait analysis laboratory. They used reflective markers for 3D video data, placed on the iliac spines of the pelvis, the malleoli, and the condyles of the knee. Patients were measured on a 7.62-m (25-foot) walkway while receiving dopaminergic therapy. They did this three times as a baseline measure: once without Listenmee^®^, once with Listenmee^®^ but without RAS, and once with Listenmee^®^ and RAS.

The results revealed significant (*p* = 0.0117) improvements in gait performance over three major dependent variables: walking speed by 38.1%, cadence by 28.1%, and stride length by 44.5%. All patients improved in the motion analysis.

Twenty patients with PD participated in the experiment of Uchitomi et al. [21] (12 women; 8 men, mean age 69.2 years). In the trial, PD patients and healthy participants walked with no auditory stimulation, a fixed-pace RAS, and an interactive RAS (Walk-Mate). The interactive system used foot sensors and nonlinear oscillators to track and reciprocate the step timing of humans. 

No difference was found for the fractal scaling exponent, the mean, and the coefficient of variation for stride intervals between the groups during the baseline walking trial (*p* > 0.1). The gait-relearning effect, based on the fractal scaling exponent of the stride interval, was significantly higher in the interactive Walk-Mate group than in the other groups (*p* < 0.05). The mean and coefficient of variation of stride intervals did not differ significantly between all the four conditions when they analyzed the interpersonal gait-relearning effect (resp. *p* = 0.3 and *p* = 0.2). The PD subjects in the fixed tempo group and the 1/f fluctuating tempo group did not synchronize their stride intervals with the rhythmic cue (*p* > 0.1). Those in the interactive Walk-Mate group did synchronize with the rhythmic cues (*p* < 0.01). In the same group, the mean of the circular variance was lower than that in the fixed tempo group and the 1/f fluctuating tempo group (*p* < 0.01 in both cases).

Zhao et al. [22] evaluated rhythmic visual and auditory cueing in a laboratory setting with a custom-made application for Google Glass. Twelve participants with PD (mean age 66.8 ± 6.8 years; 9 M, 3 F) were tested at the end of their medication dose. The subjects performed a series of walking tasks on four different walking courses while wearing the Google Glass, in combination with four cueing conditions (no cue, metronome, LED, and optic flow). For the freezing of gait analysis, they made a video recording of the walking trials. In a post hoc analysis, the number and duration of FOG during each trial was scored, and the activity (e.g., turning or walking straight) associated with each FOG episode was noted. They used a MVN motion capture suit in the lower body configuration to collect motion data. Seven MTx IMUs were attached to the pelvis, the upper legs, the lower legs, and both feet with adhesive straps. The motion data consisted of stride length, walking speed, and cadence. 

FOG occurred in a higher percentage of participants and more frequently during 360° turns compared to narrow 180° turns (*p* < 0.05), although the FOG duration did not significantly differ (*p* > 0.05). The number of FOG episodes per trial (*p* = 0.063) and the FOG duration (*p* = 0.5) were not significantly different amongst cueing conditions. However, during 360° turns, less participants experienced FOG whilst using a cue, and significantly less FOG episodes occurred per trial while using the metronome compared to no cues (*p* < 0.05). The stride length variability significantly decreased in all cues in comparison to that for no cues (*p* < 0.001). The metronome caused a significant increase in the stride length (*p* < 0.05) compared to no cues while the optic flow (*p* < 0.01) and LED (*p* < 0.005) caused a decrease in stride length. The walking speed in the doorway course was significantly faster with the metronome (*p* < 0.05) compared to no cues. During the wide turn and narrow turn course, the walking speed decreased significantly with the optic (*p* < 0.01, *p* < 0.001) and LED (*p* < 0.001, *p* < 0.001). There was a significant decrease in cadence for all cues compared to no cues for the narrow (metronome: *p* < 0.01; LED: *p* < 0.001; optic flow: *p* < 0.001) and full turn courses (metronome: *p* < 0.01; LED: *p* < 0.05; optic flow: *p* < 0.01). During the doorway course, no significant effects were found for any cue. Only the LED was associated with a significant decrease during the wide turn course in cadence (*p* < 0.01).

Thirty idiopathic PD subjects (mean age 74.9 ± 7.1 years; 14 M; 16 F) with normal hearing and no evidence of dementia participated in the study of Uchitomi et al. [30].

Eighteen healthy (mean age 70.6 ± 3.07 years; 12 M; 6 F), age-matched, non-hospitalized participants without PD who did not have any gait disorder and who also had normal hearing and no dementia formed the control group. The aim of this study was to evaluate the effectiveness of Walk-Mate on festinating gait among subjects with PD. The Walk-Mate system generated the interactive rhythmic cues and interpersonally interacted with the subject’s individual gait rhythm using rhythmic cues. It consists of a PC laptop, headphones, and pressure sensors, which are attached to the bottom of the subject’s shoes. A radio transmitter connects the pressure sensors, and a radio receiver connects the sensors and the laptop. The stride interval of each subject’s gait was provided based on the calculation of the time series of time stamps of steps in their gait. To quantify the degree of festinating gait and to determine the influence on the festinating gait of the interactive rhythmic cue generated by the Walk-Mate system, the rate of change of stride interval (β) was evaluated. The mean and coefficient of variance in stride interval were also calculated. 

Rate β was affected by the experimental conditions (*p* < 0.001). In the pre-interaction condition group, rate β was significantly smaller compared to the interaction condition group (*p* < 0.001), the post-interaction condition group (*p* < 0.001), and the control group (*p* < 0.001). However, rate β in the interaction condition group did not significantly differ from the post-interaction condition (*p* = 0.3) and the control condition (*p* = 0.06). In the control condition, it was also not significantly different from the PD post-interaction condition (*p* = 0.9). When comparing the mean stride interval among the four groups, the experimental conditions did not affect the mean stride interval (*p* = 0.8) and there was no significant difference between the means among the four groups (*p* > 0.05 for all comparisons). The experimental conditions did affect the coefficient of variation of stride interval (*p* < 0.001). The lower this value is, the more precise the estimation of the stride interval. It was significantly smaller in the control condition group than in the three other groups (*p* < 0.01). However, there were nonsignificant differences between the pre-interaction, interaction, and post-interaction condition groups within PD subjects (*p* > 0.1).

The main purpose of the Ginis et al. [20] study was to compare the effects of different types of external input between patients with PD with FOG and without FOG provided during prolonged walking. They recruited twenty-eight people with PD, categorized into FOG+ (n = 15; 14 M, 1 F; mean age 62.8 ± 6.91 years) and FOG- (n = 13; 9 M, 4 F; mean age 61.15 ± 7.08 years). All patients were tested in their subjective on-stage: on average, 1 h after they took medication. They received, in a randomized order, one of the 4 conditions (ConCue; IntCue; IntFB; NoInfo) during their entire 30-min walk. To examine the subjective preference for one of the four conditions, the participants filled in a questionnaire after the last walk. The number of gait deviations, defined as number of incidents when the mean cadence of five consecutive left and right strides, deviated more than 5% from the reference cadence, and was the primary outcome of the two foot-mounted IMU’s. 

A significant interaction effect was found between group and condition (*p* = 0.015) for the effect of condition on the number of deviations. The FOG+ group had significantly more deviations than the FOG- group during IntCue (*p* = 0.04) and IntFB (*p* = 0.03). Within FOG+, there were significantly less deviations during ConCue compared to NoInf (*p* = 0.02) and IntFB (*p* = 0.01). In the FOG- group, no significant differences were found between the conditions. Most participants preferred the IntCue and IntFB.

Thirty individuals with PD (*n* = 30 (20 men; 10 women, mean age 72 ± 6 years)) and eighteen healthy controls were participants in the study by Erra et al. [19]. This study examines the imminent impact of RAS on gait kinematics in PD ON/OFF medication. For measurements, four force resistance sensors were positioned under each foot, at the heel, first metatarsophalangeal, fifth metatarsophalangeal, and toe, to assess the temporal parameters of gait. Elastic belts were used to secure IMUs to each body segment. IMUs were used to evaluate the kinematics of the lower extremities. 

For the post-hoc analysis, they analyzed first the entire cohort of patients with PD (G1) and then the patients presenting high levels of gait impairment (G2). For G1, they found that, with RAS90, the cadence (*p* < 0.01), walking speed (*p* < 0.01), stride time (*p* < 0.01), loading response (*p* < 0.01), and flat foot (*p* = 0.02) decreased, and that the pre-swing phase increased (*p* = 0.01) significantly. With RAS100, a decrease in stride time (*p* < 0.01) and an increase in the pre-swing phase (*p* = 0.01) was found. An increase in pre-swing (*p* = 0.01) and GPQI (*p* = 0.04) was found with RAS110. In G2, RAS90 caused an increase in step length, stride length, and stride time (*p* < 0.01, in all cases). With RAS100, an increase in step length and stride length (both *p* < 0.01), and a decrease in GPQI (*p* = 0.01), was found. RAS110 decreased the stride time (*p* < 0.01) and GPQI (*p* = 0.01) and increased the step length (*p* < 0.01), stride length (*p* < 0.01), cadence (*p* = 0.05), and WS (*p* = 0.02).

Kang et al. [26] investigated the effects of no auditory (NAC), rhythmic auditory (RAC), and melodic auditory cueing (MAC) on shoulder abduction, holding, and adduction in patients with hemiparetic stroke. Kinematic data were obtained using 6 wearable bands. The Spatiotemporal kinematic parameters measured were Range of Motion, Movement Time, and Movement Variability. The wearables were placed on the head, torso, arms, and forearms. During the holding phase, MAC significantly increased the minimum Euler angle and decreased the range of motion (*p* < 0.01) compared with the other types of cueing. Further, the duration of movement execution was significantly (*p* < 0.001) shorter during the holding phase when melodic auditory cueing was provided than when the other types of cueing were used. 

While in the study of Cai et al. [31], patients walking with cooperative rhythmic stimulation had stride time fractal scaling exponents much closer to the health controls’ normal walking. The cooperative rhythmic stimulation also led to a significantly higher stride time fractal scaling exponent than the fixed-tempo stimulation condition. Subsequent questionnaires from the perspective of subjectively perceived movement stability also indicate that PD patients preferred the cooperative rhythmic stimulation. They reported that their movements with the cooperative rhythmic stimulation felt significantly more stable than with the fixed tempo of the silent controls. The fixed-tempo stimulation can improve gait impairments when the patients are instructed to synchronize.

#### 3.3.3. Music-Based Therapy and RAS

Moumdjian et al. [28] investigated if a person with Multiple Sclerosis, compared to healthy controls (HC), sustains synchronization for 12 min when they walk while listening to music, metronome sound, or absence of sound. They also investigated the effects on perceived physical and cognitive fatigue, motivation, and gait. Auditory-motor coupling and spatiotemporal gait parameters were measured during the test. They used the visual analogue scale to measure the perceived fatigue, and the Likert scale for motivation. They used D-jogger and headphones for the music. To measure the secondary outcomes, such as cadence, speed, and stride length, they used 2 wireless IMUs around the ankles and 3 OPAL wearable sensors at the height of the ankles and sternum.

In this study, 27 subjects with Multiple Sclerosis (PwMS) and 28 healthy control (HC) group patients participated. All participants synchronized to both stimuli; however, PwMS synchronized better to music compared to metronomes (*p* = 0.0005). Auditory-motor coupling decreased significantly in the last 3 min of walking to both stimuli (*p* = 0.0056). PwMS reported less perceived cognitive fatigue when walking to music compared to both metronomes and in silence (*p* = 0.0002), and perceived similar physical fatigue levels across the conditions (*p* = 0.0002). Overall, HC walked greater distances than PwMS across all conditions. The HC’s cadence, compared to the first three minutes, was slightly increased and got more into phase-locking in the music and metronome conditions compared to the silent condition, while PwMS slightly decreased their cadence towards the end of the 12 min, compared to the first three minutes.

## 4. Discussion

In recent years, music-based interventions have been increasingly investigated in the neurological rehabilitation context. With our review, we aimed to determine whether music-based therapy or RAS while using wearables influences the rehabilitation of neurological patients. We divided the results into three groups: music-based therapy, RAS, and a combination of music-based therapy and RAS. The results of all studies confirmed that both interventions mentioned in the research question can offer significant effects on gait parameters in a neurological population, using music as an auditory stimulus.

In the articles on music-based therapy [24,25,29], we noted a significant effect on the spatiotemporal gait parameters. In the three studies [24,25,29], there was a significant improvement in stride length and walking speed. When we look at the effect of RAS on spatiotemporal gait parameters, Erra et al. [19], Lopez et al. [22], and Zhao et al. [27] found that RAS increases the stride length and cadence significantly. It also increases the walking speed [19,22,27], but, if patients with PD present high levels of gait impairments, it will decrease the walking speed [19]. Two studies [22,23,24,25,26,27] from the three studies with a significant effect of RAS on spatiotemporal gait parameters are feasibility studies, which means that they have a small sample size. This makes it not possible to generalize the significant results to the PD population; thus, the results are not clinically relevant. Based on the other studies [19,24,25,29] mentioned above, we can say that an increase in walking speed and stride length and a reduction in stride time could have a great influence in the reduction of bradykinesia symptoms, relieving discomfort in performing everyday movements in daily life activities. This means that the significant results of these studies [19,24,25,29] may also be clinically relevant. When comparing the different types of cueing, using interactive cueing (Walk-Mate) ensures the best relearning effect based on the stride interval results [21,22,23,24,25,26,27,28,29,30], which is promising for the rehabilitation of the PD patient. 

Surprisingly, the effects of music therapy are independent of the population listening to the music [25,26,27,28,29,30] but are strongly related to the genre of music offered. The studies [24,25,26,27,28,29] examined these effects of different music genres on gait parameters. These music genres were diverse, ranging from classical music (andante and allegro), pop music, rock music (motivational hard rock and arena rock) to heavy metal music. As well, at times, a distinction was made between familiar and unfamiliar music. From the results, we could state that classical music resulted in a decrease of the gait parameters (stride length and walking speed), while the other music genres, for example, heavy metal, resulted in an increase in speed and stride duration. ROM was decreased by pop music (*p* = 0.005) and increased by rock (*p* = 0.009), motivational (*p* < 0.001), and heavy metal songs (*p* = 0.014). The same applies for an improvement of gait parameters when walking to familiar music. This is because individuals synchronize movements with external rhythmic signals through an inborn internal timing process [6]. This process involves several frontoparietal networks, which include auditory, premotor, and motor areas, which are interconnected through complex basal ganglia (BG), thalamo-cortical, and cerebello-thalamo-cortical motor networks [33,34,35]. In patients with neurological disease, more specifically PD, there is a loss of automaticity and rhythmicity which relies on the damage to the cerebral mechanisms that generate a regular walking rhythm [36]. RAS and music can be used to compensate for the loss of automatic and rhythmic movements in patients [37].

Compared to our study, past reviews have also examined the use of dancing and singing, as well as playing a musical instrument by oneself [38]. The systematic review of Patterson et al. [38] examined the use of dance on a neurological population, other than PD. The results of the review [38] indicated the potential for dance to be feasible for a population with neurological disorders such as stroke, MS, SCI, and Huntington’s disease to influence gait parameters and balance. A summary of results showed significant changes in spatiotemporal gait parameters, Berg Balance Scale scores, Timed Up and Go test, and six-minute walk test that were similar to or greater than those previously reported in a review of dance for persons with PD [38]. Dance therapy [39] can be an alternative exercise program with potential benefits in affecting cognition and social integration in various neurological disorders. Playing musiconeself, on the other hand, can lead to changes in the intraparietal sulcus, and this area is involved in numerical representation and operations [40,41,42].

### 4.1. Wearables

Most of the included studies [19,20,22,24,25,26,28,29] used inertial measurement units (IMUs) for the gait analysis. The validity and reliability must be considered to make a conclusion about the spatiotemporal parameters and the influence of music or RAS on them. In the systematic review of Kobsar et al. [43], they studied the validity and reliability of measuring step time, stride time, step length, stride length, stance time, and swing time while using wearable inertial sensors on healthy adults while walking. Their metrics (i.e., r, ICC) were interpreted as poor (<0.500), moderate (0.500–0.749), good (0.750–0.899), and excellent (≥0.900). They stated that step time and stride time presented the strongest body of evidence for excellent validity and reliability. Step length and stride length was found for good to excellent validity and reliability across a variety of placements (e.g., foot, shank, back). Overall, they state that their findings are supportive of the assessment of mean spatiotemporal outcomes using IMUs, but there was no difference in results due to the body placement of the IMUs. Spatiotemporal variability and symmetry outcomes (step time, step length, stance time, and swing time), on the contrary, had poor to moderate validity when measured at the back. We can conclude that using IMUs to measure the mean spatiotemporal outcomes can be done without a specific preference of the placement. To measure spatiotemporal variability and symmetry with IMUs, it is better when they are not placed at the back.

Horsley et al. [44] researched the impact of the placement of the IMUs on their validity and reliability. This study [44] demonstrated that valid and reliable derivations of stride metrics are possible from IMUs mounted on the foot, tibia, and lumbar spine. This suggests that the most critical factor may not be the location, and that validity and reliability may be more dependent on the definition of the algorithm for calculating the spatiotemporal parameters in the gait analysis.

### 4.2. Environment 

Most walking conditions were conducted in the same standardized track or environment by all participants [20,21]. This way, the participants could become accustomed to the experimental environment. 

The study of Baram et al. [45] determined that long-term treatment programs should be developed and tested in-clinic, at home, and, if possible, in a variety of natural environments to have a better outcome. This way, we have a better view on what the effects are, not only in the controlled setting, but also in more realistic day-to-day settings. For example, smart glasses can deliver external cues that may improve gait in people with PD in their natural environment. However, the potential of these devices must first be assessed in controlled experiments before they can be tested in the natural environment. The use of wearable devices makes it easier to assess the effects of music and/or RAS during daily life.

### 4.3. On or off Medication (PD)

In most of the studies, the participants were on anti-Parkinson medication [19,20,21,24,25,29]. In only two studies, the patients were off dopamine [19,27]. For the study of Zhao et al. [22], the patients were at the end of their medication dose. The effect of medication is something that must be considered to make a conclusion about the spatiotemporal parameters during the influence of music or RAS. We know that RAS was only statistically significant during the off-medication condition (Swing: *p* = 0.02, CoVSwing: *p* = 0.01). This is because PD patients in OFF condition tend to spend a longer part of the gait cycle in double support to increase stability, as previously described in another study [19]. RAS is also a cost-efficient method complementary to medication for coping with motor symptoms in PD [46]. Stride length also improved majorly using auditory cueing with Listenmee^®^ when compared to performance during the off-medication period in un-cued conditions [27]. This can also be confirmed by the study of Pedersen et al. [47] that revealed that the gait parameters which are most sensitive to anti-Parkinson medication are walking speed and stride length. 

This information [47] suggests that it is important to notify if the patients are on or off medication, as this can have an impact on the gait parameters that are measured. Medication would have an effect during off periods, as they could simply have a greater advantage. Stride length, speed, and double support in the gait cycle are mostly influenced by medication. These parameters are influenced by medication, but the question is if they will also be influenced by RAS when they are on medication, or whether this is solely an effect of the medication. Therefore, the effect of RAS can be seen limited or not significant, but this does not mean that RAS has no influence on these gait parameters during the on periods. 

### 4.4. Severity of Condition

Most of the studies that involved patients with PD used the Hoehn and Yahr (H&Y) scale to quantify their level of severity of the disease. Most patients were around H&Y stage of 2–3 [18,20,21,27]. Only in the study of Park et al. [29], patients that participated had a score between 1–2.5 on the H&Y scale. From the results, we could derive that the patients with a H&Y score between 1–3 are more viable to have positive outcomes with the use of RAS or auditory cues. It is unclear if patients with a different H&Y score would have the same improvements of gait parameters when walking with RAS or other auditory cues. 

### 4.5. Limitations

All efforts were made to ensure the methodological accuracy of this systematic review. Nevertheless, we would like to acknowledge some limitations of this study. In general, we included more studies related to PD (ten articles; [18,19,20,21,22,24,25,27,29,30] than MS (one article; [28]) or stroke (one article; ([26])). 

Although we mainly included a population with PD, it is important to form a generalization for the neurological population with movement deficits. Due to the differences in aetiology and pathology between the neurological disorders, it is not possible to do further generalized research on the four included neurological pathologies. Further research on a wider neurological population is key. This can be done separately on the different neurological populations, and, afterwards, further research can attempt to compare the similarities and or differences of the effects. However, many studies only describe PD [7,8,9], and not enough research has been done on MS, stroke, or SCI patients. Since these populations can also present themselves with motor deficits, they could potentially benefit from this research as well.

Almost all the articles [18,19,20,21,22,24,25,27,29,30] examined the participants while walking in a safe environment (e.g., a corridor, a walkway). It is possible that practice trials under different conditions would result in a different performance in locomotion outside the laboratory setting, including environmental situations such as crossing the street at a traffic light or walking outside in a busy street. Further research needs to be done on these different conditions while walking. It is therefore very essential to test the applications used in each case first in clinical settings, then as part of a rehabilitation program, and afterwards independently in daily life.

Level of cognition or level of attention could also have an impact on the results. In general, all the participants in these studies had no cognitive impairments [18,19,20,21,22,24,25,27,29,30]. Continuous cueing is less dependent on cognitive function and is possibly less attention-demanding compared to intermittent cueing. This supports the findings that traditional cueing is a useful strategy for facilitating walking in people with cognitive impairments [48].

Another limitation is that the studies [19,20,21,24,27,29,30] used indirect estimation by inertial sensors, while using their own calculation algorithm. These sensors provide an objective measure for the quantification of motion, but do not enable the direct measurement of spatiotemporal parameters. This may have resulted in the estimation of toe-off, affecting the results on two specific parameters, the stance phase and the swing phase duration, and, hence, the intrinsic fractal harmony of walking that is commonly lost in PD.

In the Moumdjian et al. [28] study, the only requirement was that patients had a diagnosis of MS for longer than one year. They did not assess the different capabilities between the patients. A scale that is used often for MS is the Expanded Disability Status Scale (EDSS) [49].

## 5. Conclusions

The main objective of this study was to examine the effect of music-based therapy RAS using wearable devices in the rehabilitation of a neurological population. The additional objective was to integrate a variety of music interventions into clinical practice as an added value. From this research, it emerged that wearable devices can potentially be used to quantify and monitor motor movements and, consequently, quantify the effect of a music-based therapy RAS in rehabilitation of neurological patients in different environments. The current findings of our study confirm the role of music-based therapy RAS as a beneficial and effective tool to implement in the health care system for rehabilitation of patients with movement disorders. Since ten of the twelve articles included in this study concern PD, we are unable to form a generalization to the other neurological disorders (MS, stroke, and SCI). However, it is likely that these results of the use of music therapy can be extended to other neurological disorders. In this regard, we would like to bring attention and indicate the need to identify standardized methods and global strategies to use music therapy in any clinical context.

## Figures and Tables

**Table 1:** Eligibility criteria following the PICOS method.

PICOSL-Question		Inclusion Criteria	Exclusion Criteria	Medical Subjects Headings (Mesh)	Free Keywords
P	Patient/population	Parkinson disease	Children	Nervous System	neurologic*, “motor disorder”, “nervous system*”, “nervous system disorder”, “nervous system disease”, stroke, Parkinson disease, Parkinson, multiple sclerosis
		Stroke patients	Healthy persons	Diseases	
		Multiple sclerosis	Other non-neurological		
		Spinal cord injuries	disorders		
			Other neurological disorders		
			Cardiac disorders		
			Animals		
I	Intervention	Music	Other interventions	Music therapy	music, rhythmic auditory stim*, rhythmic auditory cueing, music therapy
		Rhythmic stimulation			
		wearables			
C	Comparison	/	/	/	/
O	Outcome	Movement parameters			movement, motor, motion
S	Study design	/	Systematic review	/	/
L	Language	Dutch	Other languages	/	/
		English			

**Table 2:** Search strategies.

*PubMed* *29/10/2022*	((stroke*) OR ("Parkinson disease") OR (Parkinson) OR (PD) OR ("multiple sclerosis") OR (MS) OR (“spinal cord injury”) OR (SCI)) AND ((“rhythmic auditory cueing”) OR (“rhythmic auditory stim*”) OR (RAS) OR (“music rehabilitation”) OR (rhythm*) OR (rhythmic) OR (“music therapy”) OR (melody) OR (beat) OR (metronome) OR (“rhythmic auditory stimuli”) OR (“music therap*”) OR (music) OR (tone) OR (music therapy[MeSH Terms])) AND ((capture*) OR (wear*) OR (smart*) OR (intelligent)) AND ((movement) OR (motion) OR (motor*)) *Filter: human*
*Web of Science* *29/10/2022*	TS=(((stroke*) OR ("Parkinson disease") OR (Parkinson) OR (PD) OR ("multiple sclerosis") OR (MS) OR (“spinal cord injury”) OR (SCI)) AND ((“rhythmic auditory cueing”) OR (“rhythmic auditory stim*”) OR (RAS) OR (“music rehabilitation”) OR (rhythm*) OR (rhythmic) OR (“music therapy”) OR (melody) OR (beat) OR (metronome) OR (“rhythmic auditory stimuli”) OR (“music therapy*”) OR (music) OR (tone)) AND ((capture*) OR (wear*) OR (smart*) OR (intelligent)) AND ((movement) OR (motion) OR (motor*)))
*Science direct* *29/10/2022*	*(stroke) AND ((“rhythmic auditory cueing”) OR (RAS) OR (“music rehabilitation”) OR (“music therapy”)) AND (wearables)* *(("Parkinson disease") OR (Parkinson) OR (PD)) AND ((“rhythmic auditory cueing”) OR (RAS) OR (“music rehabilitation”) OR (“music therapy”)) AND (wearables)* *(("Multiple sclerosis") OR (MS)) AND ((“rhythmic auditory cueing”) OR (RAS) OR (“music rehabilitation”) OR (“music therapy”)) AND (wearables)* *(("spinal cord injurie") OR (SCI)) AND ((“rhythmic auditory cueing”) OR (RAS) OR (“music rehabilitation”) OR (“music therapy”)) AND (wearables)*
*Medline* *29/10/2022*	((stroke* or Parkinson disease or Parkinson or PD or multiple sclerosis or MS or spinal cord injury or SCI) and (rhythmic auditory cueing or rhythmic auditory stim* or RAS or music rehabilitation or rhythm* or rhythmic or music therapy or melody or beat or metronome or rhythmic auditory stimuli or music therap* or music or tone) and (capture* or wear* or smart* or intelligent) and (movement or motion or motor*)).mp. [mp=title, abstract, original title, name of substance word, subject heading word, floating sub-heading word, keyword heading word, organism supplementary concept word, protocol supplementary concept word, rare disease supplementary concept word, unique identifier, synonyms]
*PEDro* *29/10/2022*	((stroke*) OR ("Parkinson disease") OR (Parkinson) OR (PD) OR (“multiple sclerosis”) OR (MS) OR (“spinal cord injury”) OR (SCI)) AND ((“rhythmic auditory cueing”) OR (“rhythmic auditory stim*”) OR (RAS) OR (“music rehabilitation”) OR (rhythm*) OR (rhythmic) OR (“music therapy”) OR (melody) OR (beat) OR (metronome) OR (“rhythmic auditory stimuli”) OR (“music therapy*”) OR (music) OR (tone) OR (music therapy[MeSH Terms])) AND ((capture*) OR (wear*) OR (smart*) OR (intelligent)) AND ((movement) OR (motion) OR (motor*)) *Filter: human*

**Table 6:** Overview of the custom-made wearables.

Overview of the Custom-Made Wearables	
D-Jogger [28]	The D-Jogger is an adaptive music player, consisting of a software, headphones (Sennheiser, Germany), and two wireless inertial measurement units strapped at the ankles for measuring cadence and step times (iPod, Apple, USA). The software altered the tempo of the beats in the music or the ticks in the metronome to match the individualized tempo.
Walk-Mate [21]	The Walk-Mate system is a cross-feedback system, intended to realize the cross-feedback loop between the gait rhythms generated by a human and the cue rhythms generated by the system itself based on the mutual entrainment. More specifically, the Walk-Mate system provides the interactive rhythmic cues using nonlinear oscillators. Pressure sensors and transmission devices are attached to the soles of the human’s shoes, which detect the step timings while walking. The system obtains the step timings in sequence from the sensors and calculates the stride intervals of the human in real time. The system generates rhythmic cues based on the oscillatory intervals of the oscillators. The system regulates the intervals to synchronize with the human’s stride intervals. The rhythmic cues generated in this way, called “interactive rhythmic cues”, are provided from the Walk-Mate system to the human.
Google Glass [22]	Smart glasses like the Google Glass could be used to provide personalized mobile cueing to support gait; however, in its current form, auditory cues seemed more effective than rhythmic visual cues.
Listenmee^®^ [27]	The Listenmee^®^ is a device that integrates smart glasses, headphones, and a 64 GB SD card, able to produce 100 different sounds (4 special categories identified as: “environmental”, “drums”, “electronics”, and “voices”) connected to a dedicated app. The device also has statistical analyses software that can measure changes in distance and time.
BeatWalk [24]	BeatWalk is an easy-to-use, safe, and enjoyable musical application for individualized gait rehabilitation in PD. It increases “walk for exercise” duration, thanks to high observance. BeatWalk allows patients to walk outdoors with performance feedback, while listening to step-synchronized music of various genres. It also includes a smartphone application that modifies the tempo of the music to induce spontaneous mutual synchronization with patients’ gait and ankle-worn sensors.
GotRhythm [23]	GotRhythm delivers individualized music-motor therapy to stroke survivors, using a mobile phone-based app, and provides motor performance feedback. It supports a wide set of sensors to detect a variety of physical activities, including both lower and upper limb movements, as well as fine and gross motor skills. The raw sensor data is converted by the GotRhythm app into attitude angles (yaw, pitch, and roll) in real time. The aim is to match complete movement cycles (beats) and the target beats per minute (BMP), as one movement cycle corresponds to a ‘beat’. Both the target BPM and the tolerance band (the acceptable tempo range the participant can move within) are adjusted to suit the patient’s abilities.

**Table 7:** Definitions of spatiotemporal and kinematic gait parameters and their units of measurement.

	Gait Parameters	Definition
Spatiotemporal	Walking speed (WS) in m/s	Distance walked in meters per unit of time in seconds [27].
	Stride length (SL) in m	Distance covered between two successive ground contacts of the same foot.
	Stride duration/time (SD) in s	Time between two successive ground contacts of the same foot.
	Step length in m	Distance covered between two successive ground contacts of opposite feet.
	Stance phase (St) in %	The period of time when the foot under consideration is in contact with the floor.
	Pre-swing (Pre-Sw) in %	The final double support stance period which is defined from the time of initial contact with the contralateral limb to ipsilateral toe-off.
	Swing phase (Sw) in %	The period of time when the foot is not in contact with the ground.
	Double support (DS) in %	The period of time when both feet are in contact with the ground.
	Cadence in steps/min (SPM)	Number of steps per unit of time.
	Asymmetry index (AI) in %	A normalized measure of a difference between 2 quantities, expressed as a percentage difference of either their mean or their sum, whichever is defined by a user.
	Arm swing velocity (ASV) in degree/s	The maximum rotation velocity of arm swing motion [29].
	Loading response (LR) in	The initial double support stance period, which is defined from initial contact (0%) to 10% of the gait cycle.
Kinematic	Range of motion (ROM) in degrees	Passive range of motion can be defined as the range of motion that is achieved when an outside force (such as a therapist) causes movement of a joint and is usually the maximum range of motion that a joint can move. Active range of motion is the range of motion that can be achieved when opposing muscles contract and relax, resulting in joint movement.
	GPQI	A novel global index (GPQI) was used to quantify the difference in gait phase distribution.

**Table 8:** A summary of spatiotemporal and kinematic gait parameters measured across studies.

Articles	WS	SL	Step Length	SD	St	Pre-Sw (%)	Sw	DS	Cadence	AI	ASV	LR	Flat Foot	ROM Pelvis	ROM Hip	ROM Knee	ROM Ankle	ROM Shoulder	GPQI
	(m/s)	(m)	(m)	(s)	(%)		(%)	(%)	(SPM)	(%)	(°/s)	(%)		(°)	(°)	(°)	(°)	(°)	(%)
De Bartolo et al. [25],	x	x		x	x		x	x						x					
Cochen De Cock et al. [24],	x	x							x	x									
Park et al. [29]	x	x		x					x		x								
Guimaraes et al. [18]																			
Lopez et al. [27]	x	x							x										
Uchitomi et al., 2013 [21]																			
Zhao et al. [22]	x	x							x										
Uchitomi et al., 2016 [30]																			
Ginis et al. [20]																			
Erra et al. [19]	x	x	x			x	x		x			x	x		x	x	x		x
Kang et al. [26]																		x	
Moumdjian et al. [28]	x	x							x										
Hankinson et al. [23]																			
Yi Cai et al. [31]	x	x	x	x	x		x	x											
Kantan et al. [32]																			

## Data Availability

Not applicable.
